# Concepts and Approaches to Reduce or Avoid Protein Corona Formation on Nanoparticles: Challenges and Opportunities

**DOI:** 10.1002/advs.202402935

**Published:** 2024-07-08

**Authors:** Matthias Barz, Wolfgang J. Parak, Rudolf Zentel

**Affiliations:** ^1^ Leiden Academic Centre for Drug Research (LACDR) Leiden University Leiden NL‐2333 CC Netherlands; ^2^ Institut für Nanostruktur‐ und Festkörperphysik Universität Hamburg Luruper Chaussee 149 D‐22761 Hamburg Germany; ^3^ Department of Chemistry Johannes Gutenberg‐University of Mainz Duesbergweg 10‐14 D‐55128 Mainz Germany

**Keywords:** nanomedicine, nano particles, protein corona, surface modification

## Abstract

This review describes the formation of a protein corona (or its absence) on different classes of nanoparticles, its basic principles, and its consequences for nanomedicine. For this purpose, it describes general concepts to control (guide/minimize) the interaction between artificial nanoparticles and plasma proteins to reduce protein corona formation. Thereafter, methods for the qualitative or quantitative determination of protein corona formation are presented, as well as the properties of nanoparticle surfaces, which are relevant for protein corona prevention (or formation). Thereby especially the role of grafting density of hydrophilic polymers on the surface of the nanoparticle is discussed to prevent the formation of a protein corona. In this context also the potential of detergents (surfactants) for a temporary modification as well as grafting‐to and grafting‐from approaches for a permanent modification of the surface are discussed. The review concludes by highlighting several promising avenues. This includes (i) the use of nanoparticles without protein corona for active targeting, (ii) the use of synthetic nanoparticles without protein corona formation to address the immune system, (iii) the recollection of nanoparticles with a defined protein corona after in vivo application to sample the blood proteome and (iv) further concepts to reduce protein corona formation.

## Graphical abstract

Protein adsorption on nanoparticles modifies their interaction with the biological system. It depends ‐mostly‐ on the accessibility of the rigid core to proteins and can thus be reduced (or even prevented) by coating it with a dense layer of flexible hydrophilic polymers. This review discusses systematic approaches to prevent (reduce) corona formation and its “pros and cons” for nanomedicine.

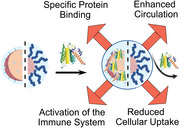

## 1 Introduction

**Figure 1:**
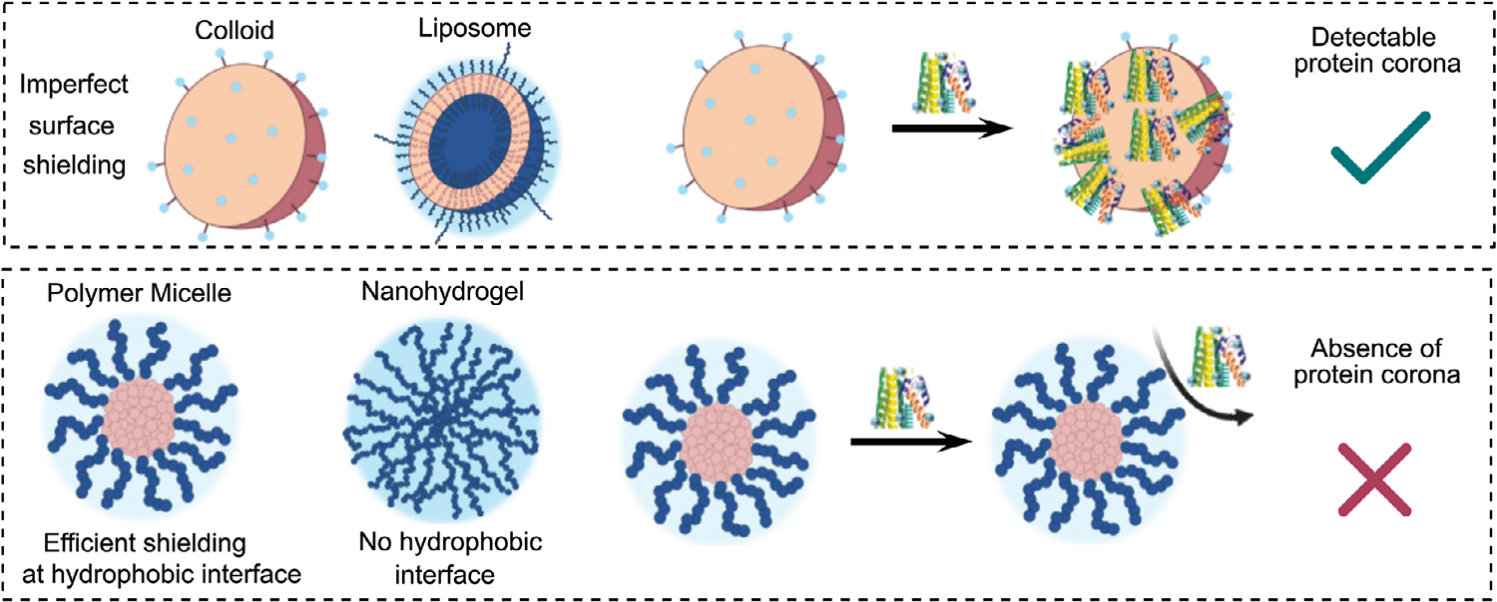
(left) Sketch of various nanoparticles (colloids, polymeric micelles, liposomes, and nanogels), which are used in the context of nanomedicines. They differ in the accessibility of their inner core (mostly hydrophobic, reddish) to proteins as outlined in ref. [86]. In typical colloids (inorganic or organic) the inner, solid core may be coated with detergents (blue). But as the solubility of the detergents in water is high, they can ‐to a certain extent‐ diffuse away from the colloidal surface enabling protein adsorption. Polymeric micelles are, however, characterized with a more stable and denser hydrophilic polymer corona than colloidal nanoparticles or liposomes. It hinders/prevents the access of plasma proteins to the hydrophobic core by entropic shielding. Liposomes, as models of cellular structures, are also not as well protected by entropic shielding (only a few long hydrophilic chains from PEG). However, their surface is covered covalently with highly hydrophilic head groups (mostly zwitter‐ionic) with pronounced hydration shell, which can reduce protein corona formation. Nanogels differ from the other systems, because they are strongly hydrated throughout the whole nanostructure and thus, they miss any interface to a hydrophobic inner core, which could act as a nucleus for protein corona formation. (right) Schematic sketch of the formation of a protein corona on purely protected nanoparticles (e.g., colloids). On the other side the hydrophilic polymer corona in core crosslinked micelles prevents the approaching of nanoparticles to the hydrophobic core.

**Figure 2:**
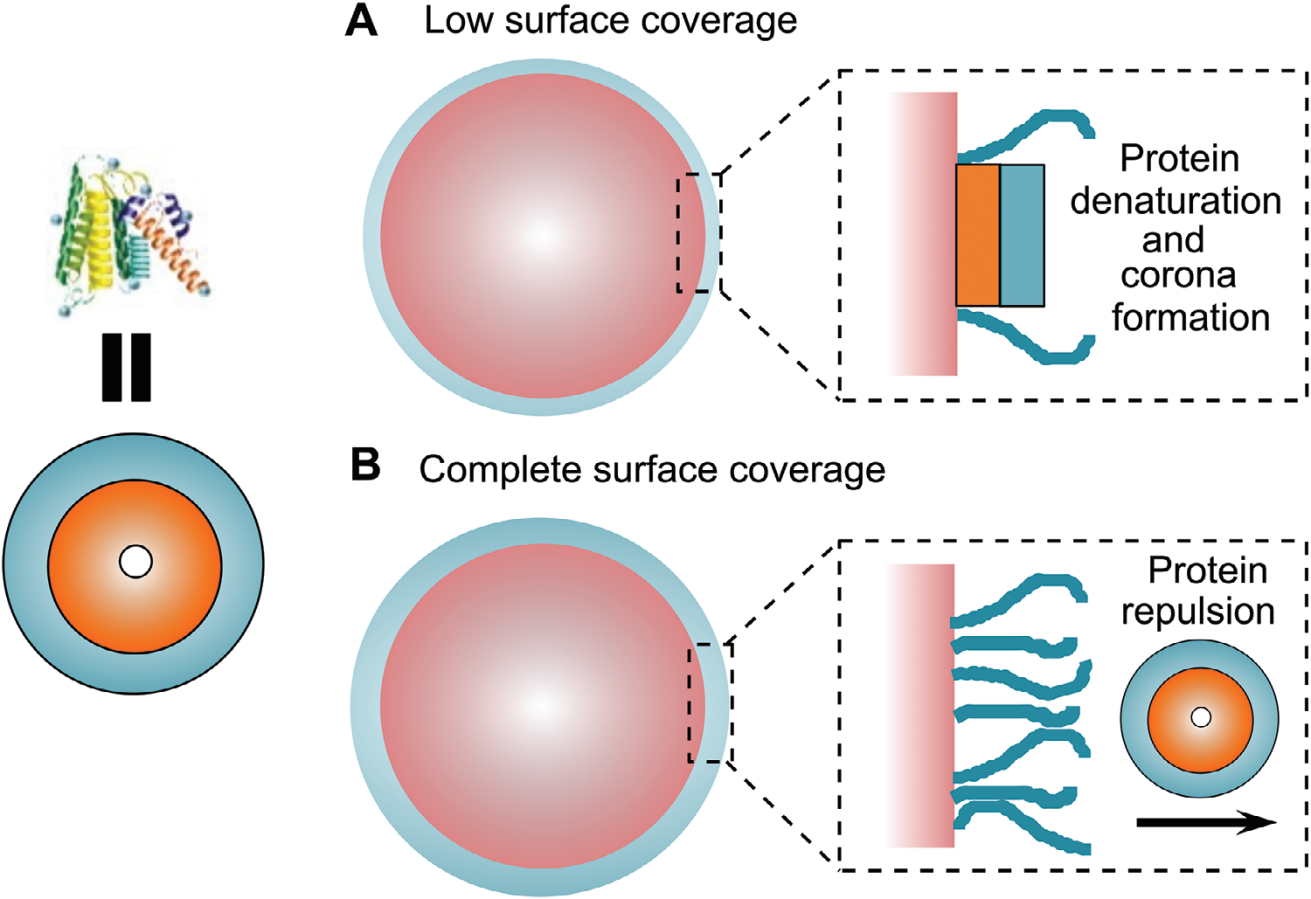
Formation of a protein corona and its prevention on surfaces; Please consider that plasma proteins tend to segregate into an inner hydrophobic core and a more hydrophilic surface (left side). So, they have an amphiphilic, colloidal character by themselves. Thus, they tend to form protein layers (often in combination with a change of their conformation) on solid surfaces, which are poorly coated (A). However, particles with a complete surface coverage (B) may avoid protein corona formation.

Over the last five decades, the world‐wide interest in nanomaterials in general and nanomedicines in particular has increased tremendously. This interest originated from the spectroscopic properties (fluorescence, plasmonic) of nanoparticles for which their nanoscopic size is most important.^[^ ^1^, ^2^ ^]^ But applications have quickly expanded to other areas, such as medicine, biology, and engineering.^[^ ^3^ ^]^ For any specific use in vivo or in the biological context interactions with molecules of live, e.g., proteins, lipids, nucleic acids, must be considered. Such interaction with the living world may also happen accidentally when nanoparticles and materials thereof have become part of our daily life. Thus, the interest in their toxicology (nanotox) and its scientific basis gained importance.^[^ ^4^, ^5^, ^6^, ^7^, ^8^, ^9^ ^]^ Within the initial studies on nanotox, researchers quickly recognized the formation of a protein layer around various nanoparticles (for various types of nanoparticles studied see **Figure** **1**).^[^ ^10^ ^]^ Therefore, protein adsorption on (in general planar) surfaces and its biological consequences have been known for a long time (see **Figure** **2**).^[^ ^11^, ^12^ ^]^ There are also reports in which early‐on the uptake of particles by cells in serum supplemented (i.e., protein containing) versus serum free medium was reported, yes without understanding of the underlying molecular mechanisms.^[^ ^13^ ^]^ However, credit must be given to Kenneth Dawson and his colleagues for being the first to comprehensively elucidate the effects of adsorbed protein layers on nanoparticle surfaces, which they termed the protein corona.^[^ ^14^, ^15^, ^16^ ^]^

In parallel to research on the bio‐interphase, several nanosized drugs and drug delivery systems have progressed through clinical trials, and received approval as drugs.^[^ ^17^, ^18^, ^19^, ^20^, ^21^, ^22^, ^23^ ^]^ In this case it is the intention, to deliver active pharmaceutical ingredients (API) to desired places in the body (e.g., to tumors,^[^ ^18^, ^19^, ^20^, ^21^, ^22^, ^23^, ^24^ ^]^ central nervous system, inflamed tissues, or to the central organs of the immune system, like the spleen^[^ ^25^ ^]^) independent of the physico‐chemical properties (e.g., solubility) of the API. An uncontrolled formation of a protein corona can be highly unfavorable in this case,^[^ ^26^, ^27^ ^]^ because it modifies the surface of the drug delivery system,^[^ ^28^ ^]^ alters pharmaco kinetics and biodistribution or ‐in the worst case‐ causes rapid nanoparticle aggregation and opsonization by immune cells. In addition, protein corona formation happens in a patient dependent manner^[^ ^27^, ^29^, ^30^, ^31^, ^32^ ^]^ (e.g., it has been observed that lean and obese animals form a different protein corona on the same nanoparticles).^[^ ^30^, ^33^ ^]^ This difference can cause significant variations of pharmacokinetics (PK) and biodistribution between patients,^[^ ^34^ ^]^ which further complicates the development of nanomedicines.^[^ ^14^, ^16^, ^30^, ^35^, ^36^, ^37^ ^]^ Besides these severe effects protein adsorption may reduce the circulation time,^[^ ^38^ ^]^ it may modify the interaction with cells and reduce specific interactions between targeting moieties and cellular antigens (shield recognition units and introduce new ones), leading to the loss of therapeutic efficacy.^[^ ^7^, ^26^, ^27^, ^39^, ^40^ ^]^

Some of the early studies on the formation of the protein corona focused on commercially available nanoparticles, which are of relevance for environmental nanotoxicology studies.^[^ ^4^, ^5^, ^10^, ^34^, ^40^, ^44^, ^45^, ^46^, ^47^, ^48^, ^49^, ^50^, ^51^ ^]^ Inorganic and organic colloidal nanoparticles (e.g., metallic, semiconducting, as well as polymeric (polystyrene) nanoparticles^[^ ^43^ ^]^) were tested first (see Figure 1). Most of these systems were and are commercial availability (certified grades) or can be easily synthesized, which eases the sample preparation workflow, incubation in serum, centrifugation and resuspensions, and protein corona analysis (gel, mass spectroscopy).^[^ ^4^, ^5^, ^10^, ^34^, ^40^, ^44^, ^45^, ^46^, ^47^, ^48^, ^49^, ^50^, ^51^ ^]^ On the other hand, in order to better understand how the physico‐chemical properties of nanoparticles can affect the formation of a protein corona specially designed nanoparticles have been prepared.^[^ ^52^ ^]^ Designed nanoparticle libraries have been used to investigate the influence of different physico‐chemical parameters such as size,^[^ ^53^ ^]^ surface polarity and charge,^[^ ^54^ ^]^ or colloidal stability.^[^ ^55^ ^]^ Unfortunately, most of these parameters cannot be varied independently, and thus, even after decades of research, it is not possible to fully predict the protein corona formation based on the nanoparticles' physicochemical properties.^[^ ^56^ ^]^

For most types of nanoparticles, a pronounced protein corona formation in contact with biological fluids can be observed. It is driven by multiple unspecific interactions, e.g. electrostatic, dipol‐dipol interactions, hydrophobic interactions, or hydrogen bonding, at the sharp surface of the nanoparticle with the plasma proteins in analogy to the formation of protein layers on macroscopic surfaces exposed to body fluids.^[^ ^12^, ^14^, ^15^, ^16^, ^57^, ^58^, ^59^, ^60^, ^61^ ^]^ Based on these results the idea arose that “every nanoparticle in contact with plasma proteins will form a protein corona” and this idea was transferred to particles to be used as nanomedicines.^[^ ^7^, ^8^, ^9^, ^10^, ^14^, ^26^, ^27^, ^62^, ^63^, ^64^, ^65^, ^66^, ^67^, ^68^ ^]^ The protein corona formation is here of particular importance, since the body will actually at first recognize the nanoparticle surface, i.e., the protein corona.^[^ ^7^, ^16^, ^26^, ^27^, ^28^, ^69^, ^70^ ^]^ Thus, prediction of nanocarrier–cell interactions is only possible, if the protein corona is taken into account.

Only recently it was recognized that protein corona formation is not happening on all nanoparticular systems^[^ ^71^, ^72^, ^73^ ^]^ and nanoparticles without significant protein corona (i.e., often less than one protein per nanoparticle, in average) are indeed accessible. This observation was, however, overlooked in the field of protein corona research, which focuses on investigations on nanoparticles for which protein corona formation occurs^[^ ^7^, ^8^, ^9^, ^26^, ^27^, ^28^, ^68^, ^74^ ^]^ and accounts for about 1.500 publications up to middle of 2022.^[^ ^7^ ^]^ It is thus the intention of this review to discuss the basis of “corona formation” or their absence and its consequences for the future development of nanomedicines.

### 1.1 Physicochemical Considerations

General consequences of protein adsorption on nanoparticles are: an increase in size and the creation of a typically negative surface charge owing to the anionic character of most blood proteins.^[^ ^26^, ^27^ ^]^

There are many different ways on how protein corona formation can be detected and different techniques may lead to different models and interpretations^[^ ^75^ ^]^ (see Section 2 and Discussion of Figure 4). Most of these methods require a purification step by which unbound proteins are removed from the solution. In situ measurements of protein corona formation are possible by quantifying the increase in size of the nanoparticles upon protein corona adsorption by various techniques^[^ ^52^, ^76^, ^77^ ^]^ (these measurements due not require purification steps). Thereafter the most popular and widespread means of analysis is mass spectrometry, which then allows to determine the protein composition of the proteins bound to the nanoparticles. Dawson and coworkers showed, that the adsorbed proteins can be divided conceptually in two parts: (i) an inner layer of irreversibly attached proteins, resisting rinsing steps, termed as the “hard corona”, and an outer layer of loosely bound and continuously interchanging proteins, which can be removed by shear flow upon rinsing, termed the “soft corona”.^[^ ^14^, ^15^ ^]^ The concept of the hard and soft corona is since then widely used in literature.^[^ ^27^ ^]^ However, there are also alternative ways of description. The adsorption of different proteins to the surface of a nanoparticle can ‐generally‐ be described in terms of respective dissociation constants (apparent K_D_ value).^[^ ^78^, ^79^ ^]^ Proteins forming a hard corona in this concept are characterized by a lower apparent K_D_ value than proteins forming a soft corona.^[^ ^78^, ^79^ ^]^

In order to modulate the amount of protein corona formed around a nanoparticle, the modulation of surface properties is commonly pursued. It is thereby the goal to work ‐on on side‐ with surfaces, which are not so attractive for proteins and on the other side shield the nanoparticle surface to prevent a direct interaction of proteins with it as far as possible. PEGylation is for example a popular way to reduce protein adsorption to nanoparticles.^[^ ^80^ ^]^ It works by preventing (reducing) the direct contact between protein and (nanoparticle or macroscopic) surface by introducing an additional hydrophilic polymer layer. Due to the curvature of the nanoparticles the molecular weight and density of the PEG coating is hereby of extreme importance (for larger particles an effective shielding is thereby easier to achieve due to the reduced surface curvature).^[^ ^81^ ^]^ More recently, zwitterionic surfaces, which get highly hydrated,^[^ ^82^, ^83^ ^]^ have also been suggested as protein repellant coating.^[^ ^84^, ^85^ ^]^ In fact, nanoparticles with zwitterionic surfaces have been reported, for which an absence of corona formation is observed within the limits of accuracy.^[^ ^71^ ^]^ Alternatively on can describe such cases, that the apparent K_D_ value of this protein nanoparticle surface interaction lies about the experimentally accessible protein concentration.^[^ ^85^ ^]^ In this case, it is assumed that approaching proteins interact ‐nearly exclusively‐ with the hydrated water layer on the surface and (nearly) not with the underlying zwitterionic material.

Therefore, protein corona formation ‐and its magnitude‐ depends on the structure of the nanoparticle surface in contact with the proteins (see Figure 1 and **Table** **1**).^[^ ^86^ ^]^ Interestingly, especially nanoparticles based on hydrophobic materials, such as carbon, latex, or polystyrene based nanoparticles, tend to form a protein corona, likely due to imperfect surface shielding/coverage.

**Table 1:** Protein corona formation on different nanoparticles (NP) like colloids, polymeric micelles (and comparable structures), liposomes, and nanogels (see Figure 1a).

NP	Properties	Refs.
Colloid 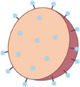	surface of the nanoparticle easily accessible; might be coated with detergents,but they can diffuse away ð Always protein corona formed	[26, 27, 34, 43, 92, 93]
Block copolymer micelle 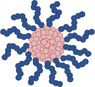	surface densely coated with a strongly hydrated,swollen but uncharged polymer brush layer:this acts as entropic cushion ð Examples without protein corona known	[44, 59, 61, 71, 91, 94, 95, 96, 98, 101]
Liposome 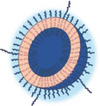	bilayer structures with an hydrophilic interior,build from lipids; strongly hydrated surface (zwitter‐ionic lipids)partial coating with hydrophilic polymers ð Mostly protein corona formation	[29, 62, 64, 99, 100, 104, 105, 106, 107, 108, 109, 110, 114]
Nanohydrogel 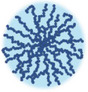	fully hydrophilic swollen structure,no hydrophilic/hydrophobic interface, no sharp interface at all; proteins may by locked‐in temporarily ð in the network structure	[94, 102, 113]

It is in this context‐ important to remember that although protein corona formation on nanoparticles happens on the nanoscale and on particles with high surface curvature, it is based on the same physicochemical effects, that govern protein corona formation on macroscopic surfaces (see Figure 2) in contact with blood plasma (e.g., in analytical devices or on needles and stents).^[^ ^57^, ^58^, ^59^, ^60^, ^61^ ^]^ And for such macroscopic substrates protein corona formation (and ways to circumvent it) has been investigated for a several decades.^[^ ^12^, ^57^, ^58^, ^59^, ^60^, ^61^, ^87^, ^88^ ^]^ Thereby,, it can be concluded that the driving force for protein corona formation is the large interface (associated with a large interfacial tension) between hydrophobic and hydrophilic regions in the “colloidal solution” of plasma proteins (most of the plasma proteins possess also large hydrophobic segments).^[^ ^7^, ^9^, ^12^, ^74^, ^89^, ^90^ ^]^ Thus, plasma proteins like to attach to hydrophobic surfaces to lower the surface tension and change their conformation to optimize the interaction (see Figure 2). The corona formation intensifies, if ionic interactions or strong hydrogen bonding add a long‐range component to hydrophobic interactions.

The surfaces of all nanoparticles possess a high surface energy (although the magnitude of this does change, see zwitterionic compounds) and is thus generally attractive for the adsorption of plasma proteins.^[^ ^26^, ^27^ ^]^ However, protein nanoparticle interaction is distance dependent. Thus, corona formation can best be hindered by preventing the direct contact of plasma proteins with the solid, mostly hydrophobic (nanoparticular or macroscopic) surface. This can be effectively done by coating the hydrophobic surface with a dense, swellable, hydrophilic polymer corona (see Figures 1 and 2), which prevent a direct contact of the plasma proteins with the surface. The shielding against proteins works effectively, if the density of the hydrophilic polymer corona is high (see Section 3.2. for a quantitative discussion).^[^ ^44^, ^58^, ^59^, ^60^, ^61^, ^91^ ^]^ Following this route it has been possible to prepare surfaces, which are not coated by a protein corona. To optimize the system, some aspects have to be considered.

At first, the surface of the inner part of the nanoparticle ‐ideally‐ shall possess limited attractive forces for proteins. It should be (overall) uncharged, a weak H‐bond donor, and not too hydrophobic. Thus, it will be very difficult to prevent a plasma corona formation on, e.g., polystyrene nanoparticles (pure hydro‐carbon, very hydrophobic).^[^ ^27^, ^34^, ^43^, ^92^, ^93^ ^]^ On the other side zwitterionic structures/polymers.^[^ ^72^, ^73^, ^82^, ^83^, ^91^ ^]^ are intensively discussed to achieve this. Due to their ionic structure with zero net‐charge they form a layer of strongly hydrated water molecules at their interface, so that plasma proteins ‐approaching the surface‐ interact mostly with the hydrated water.

To reduce the influence of particle core properties further it is most important to coat the surface of the nanoparticle with a dense layer of hydrophilic, swollen polymer chains. These polymers shall ‐of course‐ not interact by themself with the proteins and are thus described as protein resistant. To achieve this, all polymer chains shall be also well hydrated. Under these conditions the plasma proteins (which are hydrated themselves) will not see the hydrophilic polymers directly, but only their hydrate shells, which can prevent a direct interaction. In addition, this effect has to be complemented by an entropic repulsion of the anisotropically swollen polymer system (this aspect is most important) against proteins. Any protein approaching the nanoparticle will compress the stretched chains (lower their conformational freedom or reduce their entropy).^[^ ^59^, ^61^ ^]^ As a result this hydrophilic polymer layer will repel approaching plasma proteins by repulsive entropic forces (no attractive interactions needed) from the surface. This is known as entropic cushion.^[^ ^94^, ^95^, ^96^ ^]^ It is the same principle as providing colloidal stability to nanoparticles by steric repulsion. This concept works very efficiently on macroscopic and nanoparticular surfaces (see Figures 1 and 2).^[^ ^58^, ^61^, ^87^, ^88^ ^]^ Recent work has shown that this “entropic shielding” is not only valid for “surface grafted polymer brushes”, but also for hydrophilic crosslinked and then highly swollen polymer networks.^[^ ^87^, ^88^ ^]^

It is thus not unexpected that a recent study, which investigated and quantified the protein corona formation in core‐crosslinked polymer micelles and bottlebrush polymers coated with a dense hydrophilic polymer corona (PEG, PHPMA or pSar^[^ ^71^ ^]^) observed no protein corona formation. The amount of proteins associated to the nanoparticle fraction was quantified to be on average less than 0.6 human serum albumins (HAS) per nanoparticle. Other proteins can be identified but are far less abandoned.^[^ ^71^ ^]^ (note: Control experiments proved that the HAS was only coeluting.)^[^ ^45^, ^77^ ^]^ The other proteins, which were accumulated, accounted only for 0.1 proteins per nanoparticle; see discussion to Figure 5). In turn, these results clearly demonstrate that the investigated polymeric micelles and cylindrical bottlebrush polymers did not attract a protein corona. This happened because the nanoparticles surface is coated by a dense layer of hydrophilic protein‐resistant polymers.

This study included the CriPEC CCPM platform, which is in clinical phase 2.^[^ ^97^ ^]^ Thereby these particles showed a neglectable variation of pharmaco‐kintetics (PK) between different patients, which underlines the potential of an absence of patient specific protein corona formation.^[^ ^21^, ^71^, ^97^ ^]^ For the other systems included in this study, PK variations in mice/rats were also neglectable.^[^ ^71^ ^]^ All these systems displayed the absence of a protein corona formation, or − more quantitatively − the majority of all nanoparticles are not associated with a single protein.^[^ ^71^ ^]^ This demonstrates that the immediate formation of a significant protein corona is not a general property of all nanosized objects, which get in contact with plasma proteins, but can be controlled by the chemistry of the nanoparticles.

**Figure 3:**
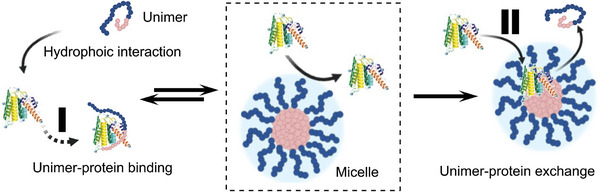
Self‐assembly of amphiphilic block copolymers into polymer micelles (compare Figure 1) ‐as well as‐ their interaction with plasma proteins. If the inner core is shielded by a dense corona of hydrophilic and protein‐resistant polymers, attractive interaction sites, e.g., the hydrophobic core, are not accessable for plasma proteins. For micelles, however, the equilibrium between micelles (middle) and unimers (left) is of major importance. It depends on the critical micelle concentration. Individual unimers are of amphiphilic nature and therefore can interact directly with plasma proteins in solution (see left, I). Alternatively an exchange reaction between unimers and proteins can occur leading to the integration of proteins into polymer micelles (see right, II).

It is thus reasonable to assume that the formation of a protein corona and its amount or ‐on the other side‐ its absence primarily depends on surface structure and especially on the accessibility of the hydrophobic interface of the nanoparticle to proteins as outlined in ref. [86] and shown in Figure 1. Therefore, we will differentiate here between “classical/typical colloids”, in which the surface is well accessible and difficult to passivate for interactions with proteins and block copolymer micelles as a model for systems, in which the surface is densely coated with a hydrophilic polymer (see Figure 1 and Table 1). In the typical colloids (nanoparticles) the sharp interface to the (mostly) hydrophobic core is easily accessible to plasma proteins. The access to the hydrophobic core of a block copolymer micelle or a bottlebrush polymer is, however, effectively hindered, if it is densely coated with a hydrophilic, strongly hydrated polymer corona, which has no “net charge” (long rang interactions). This highly swollen polymer film repels (as in the case of planar systems, Figure 2) approaching plasma proteins by repulsive entropic forces without the need of any attractive interactions.^[^ ^94^ ^]^ Such a situation is effectively achieved in core‐crosslinked block copolymer micelles or cylindrical bottlebrush polymers (see Figure 1).^[^ ^71^ ^]^ Block copolymer micelles contain a dense hydrophobic (water insoluble) core, which is surrounded by a hydrophilic polymer corona, as each hydrophobic polymer block is linked to a hydrophilic block in the “unimer” (see **Figure** **3**). Therefore, these systems possess a high density of hydrophilic chains per area at the hydrophobic/‐philic interface of the self‐assembled micelles. And if such a structure is core‐crosslinked, it can ‐no longer‐ disassemble with time^[^ ^98^ ^]^ (Figure 3) and it represents a stable core‐shell nanoparticles^[^ ^99^ ^]^ with a dense hydrophilic polymer layer around the hydrophobic core. The stability of this structure is thereby important to exclude protein corona formation with time^[^ ^98^ ^]^ as the structures rearrange (see Figure 3). Also polymeric brushes, in which hydrophilic side chains from polysarcosine are liked to each unit of the hydrophobic polymer backbone form similar core‐shell structures.^[^ ^71^, ^100^, ^101^ ^]^

Concerning further nanoparticles for biomedical applications liposomal aggregates and nanogels^[^ ^102^ ^]^ shall be briefly mentioned (see Figure 1a).

Liposomes are lipid‐based nanoparticles with a hydrophilic interior (see Figure 1). They resemble cellular or sub‐cellular structures and as lipids possess a much lower critical micelle concentrations (CMC) than detergents, their aggregates do not dissolve in water. Liposomal formulations of APIs became potent drugs (e.g., Doxil).^[^ ^103^ ^]^ Historically, corona formation on liposomes in the presence of biological fluids has also been studied.^[^ ^29^, ^62^, ^64^, ^99^, ^100^, ^104^, ^105^, ^106^, ^107^, ^108^, ^109^, ^110^ ^]^ Concerning their molecular composition liposomes are mostly prepared from natural lipids (usually in their zwitter‐ionic, i.e., macroscopically uncharged forms) and cholesterol. And because of the zwitter‐ionic structure of their surface, they are covered by a tight hydration shell,^[^ ^72^, ^73^, ^82^, ^83^ ^]^ which limits the attractive interactions with plasma proteins. This is obviously an adaption of the natural cellular membranes and also cells of the body want to prevent unintended protein corona formation on their surface. Liposomal formulation can be shielded further against protein corona formation by adding polymer modified lipids (PEGylated or pSarcosinylated lipids),^[^ ^95^, ^111^ ^]^ which add additional entropic shielding.^[^ ^29^, ^62^, ^64^, ^67^ ^]^ However the polymer density and thus the steric stabilization is usually smaller than in block copolymer micelles,^[^ ^62^ ^]^

Because entropic shielding depends strongly on the density of the hydrophilic chains.^[^ ^44^ ^]^

For liposomal formulations protein corona formation is observed by a size increase in contact with serum proteins.^[^ ^112^ ^]^ Its composition was detected by mass spectroscopy.^[^ ^29^, ^62^, ^64^, ^67^ ^]^ Due to the dynamic structure of liposomes (see Figure 3 and ref. [98]) liposomes find interest as nanoparticles, which accumulate rare proteins associated with diseases in their corona.^[^ ^31^, ^32^ ^]^

Although a hard quantitative comparison concerning the amount of protein corona formed is not possible yet (this will also depend on the lipid composition) liposomes seem to be intermediate between core‐crosslinked polymer micelles and hydrophobic colloids, e.g. polystyrene colloids.^[^ ^92^ ^]^

Nano(hydro)gels are another class of nanoparticles, which find increasing attention in nanomedicine, especially for nucleic acid and protein delivery.^[^ ^94^, ^113^ ^]^ They possess no internal sharp core‐shell structure and especially no sharp transition between a hydrophilic and a hydrophobic compartment.^[^ ^94^ ^]^ Thus, they cannot form protein layer on the surface (as there is no “dense surface”). Recent experiments demonstrated that such nano(hydro)gels absorb none or only very little plasma proteins (only passive diffusion into the hydrogel structure), when they are made from highly polar and stealth‐like polymers.^[^ ^102^, ^114^ ^]^ As they miss the amphiphilic structure they will not be discussed here further. But depending on the pore size of the gel, proteins may “accidently” diffuse into the hydrophilic network and stay there for some time.^[^ ^94^ ^]^

## 2 Analysis of the Protein Corona on Synthetic Nanoparticles

### 2.1 Identification and Quantification of the Protein Corona: How Much Protein and Which Ones Associate with Individual Nanoparticles?

Most often, the protein corona is analyzed by incubation of nanoparticles in biological fluids like blood plasma, serum, lymphatic fluids, or full blood followed by separation of the nanoparticles from non‐bound proteins,^[^ ^115^ ^]^ though in situ methods without the need for purification exist as well.^[^ ^52^, ^76^ ^]^ Purification can be done by centrifugation/resuspension, spin filtration, magnetic separation, or asymmetric flow field flow filtration (AF4). Thereafter the proteins can be analyzed by gel electrophoresis and/or LC mass spectroscopy.^[^ ^34^, ^39^, ^47^, ^62^, ^63^, ^65^, ^66^, ^71^, ^102^, ^115^, ^116^, ^117^, ^118^ ^]^ Gel‐electrophoresis in combination with LC‐mass spectroscopy allows thereafter the determination of tiny traces of proteins associated with the nanoparticle.^[^ ^115^ ^]^ With this method, it was detected that the proteins primarily adsorbed are usually apoproteins (ApoE), clusterine, and other amphiphilic proteins,^[^ ^87^, ^88^, ^115^ ^]^ which can easily adsorb to the nanoparticle with their hydrophobic units to lower the surface energy of the corresponding nanoparticle by covering remaining hydrophobic patches.

From our point of view, it is of particular importance to go beyond a characterization of the protein corona composition, and quantify the total amount of nanoparticle associated proteins,^[^ ^71^ ^]^ since the amount of protein will largely differ between different nanoparticles and cover an enormous range from a very few proteins (or less than one) per nanoparticle to a pronounced protein corona of several hundreds or thousands of different proteins. In the last case, the process of protein corona formation can be so prominent that the overall size of the nanoparticles increases strongly and even macroscopic aggregates are formed,^[^ ^119^ ^]^ which can – in the worst case‐ cause a severe embolism (lung targeting to lung embolism). This ‐in particular‐ may occur with nanoparticles of low colloidal stability, where already slight perturbations may lead to nanoparticles agglomeration. A small amount of adsorbed proteins may, on the contrary, just modify the body distribution^[^ ^101^ ^]^ and act analogously to the biological process of predesigning proteins (or biological nanoparticles) for degradation in the body (e.g., in the liver). Of note, when changes in the distribution in the body of rodents or men, often also referred to as in vivo biodistribution, are reported it is of particular relevance to quantify these changes precisely.^[^ ^56^ ^]^ Effects related to the total amount of proteins adsorbed per nanoparticle can alter the biological fate of nanoparticles more substantially than the overall composition of corona proteins.

In the first case (large amount of protein corona, onset of agglomeration), corona formation can be directly detected by physico‐chemical methods, which determine the size increase of the nanoparticles (and these methods do not need a purification step). Thus, dynamic or static light scattering, fluorescence correlation spectroscopy, but also single object characterization methods, such as transmission electron microscopy (TEM) or nanoparticle tracking analysis (NTA), became standard techniques in the field.^[^ ^39^, ^62^, ^63^, ^65^, ^66^, ^100^, ^115^, ^116^, ^119^, ^120^, ^121^ ^]^

In the case of nanoparticles with a prominent protein corona, Landfester and coworkers determined, e.g., the amount of adsorbed proteins on polystyrene latex particles (diameter: 170 to 200 nm) to be 350 to 1330 proteins per nanoparticle (depending on the functionalization and the pH) by isothermal titration calorimetry.^[^ ^92^ ^]^ The associated protein corona could be visualized by TEM measurements.^[^ ^93^ ^]^ Also for hydroxyethyl starch (HES) nanocapsules, which are prepared by dense crosslinking in an inverse miniemulsion (note: due to this process they can be considered to be more like a hollow sphere, containing detergents and they are not sterically stabilized toward the outside!) similarly high amounts of adsorbed proteins were observed.^[^ ^116^ ^]^ This presents a huge contrast to the results of the studies for core crosslinked micelles or cylindrical bottlebrush polymers,^[^ ^71^, ^98^ ^]^ where less than one protein molecule per micelle was found.^[^ ^71^ ^]^ Size measurements are however not always straight‐forward. While agglomeration in general always can be conveniently detected, a significant size increase may require more than the absorption of some proteins. Especially, for DLS, FCS, and NTA a sufficient percentual size change is required. Adsorption of only one serum albumin protein will lead to a drastic increase in size of a nanoparticle with 2 nm hydrodynamic radius,^[^ ^122^ ^]^ but would no longer lead to significant size increase for nanoparticles of 50 nm and above. Size measurements on small nanoparticles with good size distribution can be very powerful analytical tool to detect small amounts of proteins, but they do not allow to tell WHAT proteins have adsorbed (for this mass spectrometry needs to be employed), and the range of operation is limited to sizes below 50 nm. For inhomogeneous nanoparticles with a broad size distribution, the absence of an increase in hydrodynamic radius in the presence of proteins does not necessarily indicate the absence of a protein corona.

Due to their remarkable sensitivity, mass spectrometry will, however, always detect some proteins in any sample, although this can well result from imperfect purification or contaminations. As result there is a lack of information for nanoparticles with diameters above 50 nm and a small (or no) tendency for corona formation. However, these nanoparticles are especially interesting for biomedical applications in vivo or *ex vivo*. For example for water‐ soluble polymers by themselves, for core crosslinked micelles, for nanogels or liposoms the amount of plasma proteins per nanoparticle can be rather low.^[^ ^29^, ^64^, ^71^, ^102^, ^108^, ^109^, ^110^ ^]^

Gel‐electrophoresis in combination with LC‐mass spectroscopy allows nowadays the determination and quantification of traces of proteins associated with nanoparticles (that means: in the fraction of the nanoparticle after the separation method applied).^[^ ^115^ ^]^ With this method it was detected that the proteins primarily adsorbed are usually apoproteins (ApoE), clusterine, and other amphiphilic proteins.^[^ ^87^, ^88^, ^115^ ^]^ They contain hydrophobic parts and are used in the body, e.g., for the transport of lipid droplets (this will also be discussed in combination with the formation of a corona from biomolecules).^[^ ^65^, ^118^ ^]^ For some nanoparticles (mostly liposomes), which could be recovered after circulation in the living body, it was ‐in addition possible‐ to detect the host dependent integration of specific proteins.^[^ ^29^, ^31^, ^32^, ^64^ ^]^ The amount of proteins identified by mass spectroscopy is, however, usually not quantified against a reference sample. Thus, it was possible to keep the claim of an omnipresent protein corona for nanoparticular systems, which showed no size increase, even if there may be only a few proteins ‐or less than one‐ per nanoparticle.

**Figure 4:**
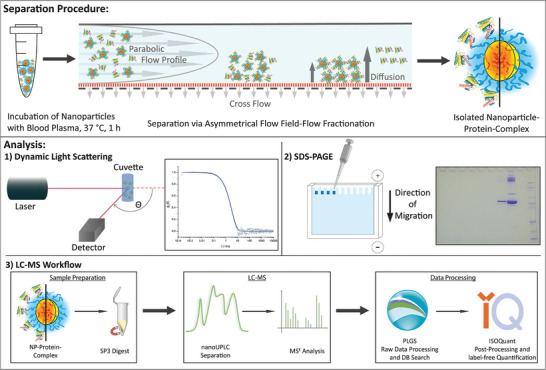
Workflow to identify type and amount of nanoparticles associated proteins. It starts with a separation step, which separates aggregates, nanoparticles, and proteins according to size (or mass) and allows to isolate the fractions (like AF4, upper line). Analysis can then be done by combining dynamic light scattering (size) and electrophoresis (separation and quantification of the proteins). At last LC‐MS allows the identification of the proteins and their relative amounts to distinguish really accumulated proteins from proteins, which are just “co‐eluting”. Reproduced with permission.^[^ ^71^ ^]^ Copyright 2020, Wiley‐VCH GmbH.

It is thus important to note that the amount of proteins on nanoparticles can be determined in a quantitative manner^[^ ^71^, ^98^ ^]^ according to the work flow presented in **Figure** **4**. The nanoparticles were ‐after incubation in blood plasma‐ separated by AF4 chromatography, which allows ‐at the same time‐ to determine if their size varies due to corona formation. The isolated fractions are then analyzed by dynamic light scattering and traces of proteins associated with nanoparticles (that means: located in the fraction of the nanoparticle after AF4 separation applied) were detected by gel electrophoresis and quantified in relation to a known internal standard. At last, nanoparticle associated proteins can be identified by LC‐mass spectrometry.^[^ ^71^, ^98^ ^]^

**Figure 5:**
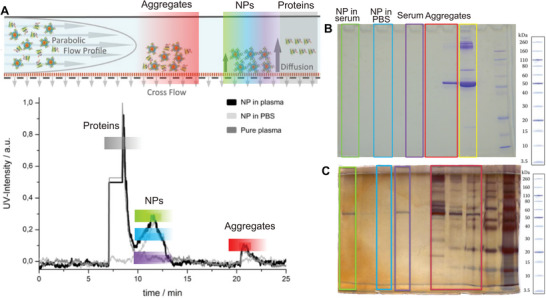
Results of the separation of nanoparticles from plasma proteins (core crosslinked polymer micelles and polymer brushes from ref. [71]) according to size by AF4 (A) (see Figure 4 for the complete workflow) and detection of proteins in the different fractions (B: Coomassie staining and C: silver staining).

For the nanoparticular systems studied there (core crosslinked polymer micelles and a cylindrical bottlebrush polymers^[^ ^71^ ^]^) no sign for the association of any protein was observed. Also, Coumassie staining (the standard method to determine proteins in gel electrophoresis) of the gels gave no evidence for any associated proteins (see **Figure** 5B). However, with silver staining, which can detect nano‐grams of protein, some proteins could be detected (see Figure 5B,C). It proved later that there were traces of serum albumin, which were coeluting with the nanoparticles. But this also opened the possibility to determine an “upper limit” for the “possibly associated” proteins, by adding increasing amount of pure serum albumin to the gel and determining, when a comparable signal appears as for the nanoparticle fraction. And as the molecular brush polymers ‐used in this study‐ were a well‐studied molecular structure of known molecular weight,^[^ ^71^ ^]^ it was possible to determine (from the masses added) that there was less than one serum albumin eluted per polymer brush (and from this, most was just coeluting and not associated). This excludes already anything like a protein corona. To identify even smaller amounts of other proteins, LC‐mass spectroscopy can be employed.^[^ ^71^ ^]^

However, because of the high sensitivity of this analytical tool, careful positive and negative controls were needed to eliminate proteins, which are simply coeluting or contaminations, to identify plasma components that qualify as a true component of the protein corona. The amount of such proteins amounted, however, only to about 0,1 protein per nanoparticle; or 90% of the nanoparticles, recovered by AF4, were not associated with any plasma protein.^[^ ^71^ ^]^ That means that the extremely small loading with proteins is inhomogeneous.^[^ ^26^ ^]^

The AF4 method (Figure 5A) separates the fractions according to size. Under these conditions small plasma proteins are eluted first, then the artificial nanoparticles (here 20 to 30 nm) with sizes larger than 10 nm and then even larger proteins or aggregates (not shown here). At last larger objects are detected in the “so called” rinsing peak, after the separation run is finished. The AF4 runs (5A) show that the artificial nanoparticles can be well separated from the plasma proteins and that their size is unchanged (within the limits of the separation method). Then the fractions ‐eluted at the time of the nanoparticle elution‐ are examined for plasma proteins (this also includes the pure plasma sample to determine the quality of the separation process). Thus Coomassie staining (5B) (standard) and the very sensitive silver staining (5C) (nanomolar) are applied to the AF4 fractions; green: fraction of the nanoparticle after plasma incubation; blue: nanoparticle in PBS buffer (no contact with plasma); purple: fraction from a pure plasma sample (no nanoparticles) under the conditions of nanoparticle separation; red: rinse peak, yellow: pure plasma, diluted to 1% (red and yellow must contain proteins). While with Coomassie staining no proteins are detectable in the nanoparticle fraction (green) and the pure plasma sample (purple), silver staining detects some serum albumin. Data taken from ref. [71].

## 3 Nanoparticles in Water and their Stabilization: The Influence of Detergents and Hydrophilic Surface Structures on Protein Corona Formation

### 3.1 Detergents and Amphiphilic Structures

At first, why do colloids so easily acquire a protein corona? Colloids consist mostly of inorganic materials like metal oxides or metals or organic materials, e.g., polystyrene, polylactide, polyglycolide, etc., which are hydrophobic. Therefore, the adsorption of endogenous proteins can lower the surface energy of NPs in aqueous solution, which drives protein adsorption. In addition, the hydrophobic surfaces usually require the use of low molecular weight or polymer surfactants or even polymer coating to enable dispersibility/solubility in aqueous solution. It is ‐in this context‐ however important to remember that ligand free nanoparticles exist (also in water), for example by laser ablation.^[^ ^123^ ^]^ They will, however, in contact with proteins be immediately coated with a protein corona.^[^ ^123^ ^]^

Common surfactants to stabilized colloidal systems in water, such as sodium dodecyl sulfate (SDS) introduce in addition charges to the colloidal surface, but do not lead to a dense polymer layer able to perform entropic shielding. Moreover, these charges enable further ionic interactions (electrostatic or entropic (salt pair release)) with proteins, which fosters protein corona formation.

Now there are also “non‐ionic detergents”, in which the hydrophilic part consists of a hydrophilic oligomer (or short polymer) like Tween or pegylated block copolymers with polylactide as hydrophobic block.^[^ ^44^, ^124^, ^125^ ^]^ It seems possible to use such detergents for the creation of a more or less dense hydrophilic shell on the surface of colloidal nanoparticles. However, all those types of detergents possess a high CMC (range of 10^−2 ^mg mL^−1)^, which indicates high exchange dynamics. In other words, there will be always free amphiphilic unimers in the solution (see Figure 3 for which detergents display an extreme case, with a very high amount of unimers), and it may cause adverse effects. Besides, these dynamics may also (i) cause the formation of hydrophobic patches on the surface of the colloid for possible protein adsorption or (ii) these unimers may interact directly with plasma proteins in solution, making an integration into the nanoparticle easier afterwards. In addition, detergents may even induce the denaturation of plasma proteins, which can trigger pronounced immune responses.^[^ ^27^, ^68^ ^]^ Since the CMC of various non‐ionic detergents (e.g., Tween), which “look” attractive for entropic shielding, is rather high, they are thus of limited applicability for the shielding of colloidal surfaces].^[^ ^10^, ^62^, ^63^, ^65^, ^119^ ^]^

This inherent complexity of interactions differentiates colloidal systems from many other types of synthetic nanoparticles, such as polymeric micelles, water‐soluble polymers by themselves, or liposomal systems.^[^ ^104^, ^105^, ^106^, ^107^ ^]^

An alternative approach to coat colloids with a dense, uncharged, but hydrophilic corona to obtain “entropically shielded” structures, could be the use of amphiphilic structures with a very low CMC (see Figure 3 with the equilibrium shifted to the stable micelle). With their help hydrophobic inorganic nanoparticle cores can be stabilized^[^ ^126^ ^]^ up to a point that after removal of excess polymer the coating remains around the nanoparticle, i.e., does not dissociate, as verified for example by TEM with negative staining.^[^ ^127^ ^]^ Amphiphiles can be made for example with amphiphilic block copolymers, e.g., with pHPMA and p‐laurylmethacrylate blocks.^[^ ^113^, ^128^ ^]^ And as they can be synthesized with a very low CMC (range from 10^−5^ to 10^−4 ^mg mL^−1)^, they can also be processed into stable thin Langmuir films on the air water interface (a monolayer, which does not dissolve in the subphase).^[^ ^129^ ^]^ More about such systems will be discussed under future directions (see below).

### 3.2 Grafting Approaches to Coat the Surface

On the other side: to protect the hard, inner part of a colloid against the access of proteins (entropical shielding), a permanently bound dense corona of hydrophilic polymers on the surface should be sufficient (see Figure 1). There are more general approaches to achieve such a dense polymer layer besides the use of block copolymers on colloids or the use of stabilized polymer micelles. Chemical approaches include grafting‐to and grafting‐from processes. The grafting‐from approach, leading to amphiphilic polymer brushes is able to prevent protein corona formation as shown for some pepto‐brushes.^[^ ^71^, ^100^, ^101^ ^]^ though from very small nanoparticles radius‐of‐curvature effects may lead to protein penetration into the brushes.^[^ ^81^ ^]^ On the other side, a systematic study reported that grafting hydrophilic polymers densely onto the surface of polystyrene colloids,^[^ ^118^ ^]^ was in this case not able to produce colloids, which avoid protein corona formation in plasma. It is not possible to determine exactly, why this grafting‐to approach was not successful, but the following data are available. According to ref. [118] the grafting of hydrophilic polymers to the interface had lead to a surface density of one hydrophilic chain per 10 nm^2^ surface (calculated from the amount of grafted hydrophilic polymer and the overall surface area of the colloids). For the self‐assembled block copolymer micelles, however, a surface coating with an higher density (about doubled) with only 5 to 6 nm^2^ per hydrophilic chain (see ref., ^[^ ^71^ ^]^ Supporting information for calculation) had been determined. So, in both systems the hydrophobic core is coated with hydrophilic polymer chains, but their density is substantially different. Interestingly the range of grafting densities for both systems, which prevent corona formation (block copolymer micelles^[^ ^71^ ^]^) or still allow it (grafted polystyrene colloids), lies in the range from only 5 to 10 nm^2^ per hydrophilic chain, and in this range the interaction of entropically shielded colloids with plasma proteins has been reported to change drastically.^[^ ^44^ ^]^ Densely coated systems (5 nm^2^) circulate very long in the blood stream, while a reduction of the grafting density (10 nm^2^) reduces the circulation times strongly. So it may be that the grafting‐to process used in^[^ ^118^ ^]^ was just a bit too inefficient to achieve the high surface density needed to shield the interface perfectly.

Grafting‐to is also a good method to coat inorganic nanoparticles with a dense layer/corona of hydrophilic polymers (entropical shielding), as there are efficient anchor groups for the inorganic core available.^[^ ^130^, ^131^ ^]^ In such a way it was possible to prevent the formation of a protein corona on iron‐oxide nano‐particles.^[^ ^130^ ^]^ For this purpose, the combination of a high grafting density and a special structure of the hydrophilic polymers (ring systems) was used.^[^ ^130^ ^]^

## 4 Future Directions: Therapeutic or Diagnostic Applications

Corona formation, its composition and especially its amount has great implication for any use of nanoparticles in the biomedical context. Nanoparticles with a high tendency for agglomeration, e.g. upon exposure to proteins, should ‐for the sake of the test animals‐ not be used at all. Otherwise, particles with some tendency for corona formation can be highly useful analytical tools.

The fact that some inorganic colloids and liposomal preparations can be recovered and analyzed after in vivo circulation^[^ ^26^, ^27^, ^29^, ^64^ ^]^ allows to enrich specific proteins and thus enables a more sensitive detection.^[^ ^29^, ^64^ ^]^ This concept allows the detection of disease related biomarkers, monitor diseases progression or therapeutic success.^[^ ^26^, ^27^ ^]^

On the other hand, the fact that there are nanoparticles without protein corona formation opens many other diagnostic or therapeutic possibilities.

### 4.1 Potential of Nanoparticles Without Detectable Protein Corona

At first such nanoparticles are very important as basis for active targeting, because only nanoparticles without a significant protein corona make a systematic, willingly functionalization (bioconjugation) to achieve active targeting of nanoobjects sensible. The ability of active targeting may not only get lost by coverage of ligands with adsorbed proteins, but an unintentionally formed protein corona can itself cause multiple undesired interaction with recognition sites in the body.^[^ ^14^, ^16^, ^26^, ^27^, ^35^, ^36^, ^37^ ^]^ Its composition is, however, hardly predictable^[^ ^29^ ^]^ and differs strongly between patients and their status at a given time.^[^ ^30^, ^33^, ^67^, ^132^ ^]^ Targeting will thus require a personalized optimization of the protein corona, which will complicate or even block clinical use.^[^ ^26^, ^27^ ^]^ Properly designed nanoparticles can solve this problem easily. As an example, nanoparticles like CPC634, which do not have a detectable protein corona, show enhanced circulation times − per se − and very little variability between the patients.^[^ ^21^, ^71^, ^97^ ^]^ Such systems can be the basis to target special cells and tissues within the body.

A topic, where active targeting will be very relevant is the selective activation of the immune system with nanoparticles.^[^ ^25^, ^133^ ^]^ This requires a preferred targeting to dendritic cells as sub‐cells of the immune system^[^ ^25^, ^101^, ^134^, ^135^ ^]^ and the delivery of alarm signals and antigens,^[^ ^25^, ^133^, ^134^ ^]^ a task, which requires nanoparticles as host for both. In this context the formation of a “spontaneous” protein corona would be very unfavorable for two reasons. At first it might reduce the active targeting.^[^ ^136^ ^]^ But, in addition it will lead to a co‐presentation of (partly denatured) body proteins (protein corona) and immune activator to very potent immune cells. Thus, a potential risk for severe auto‐immune responses needs to be considered.^[^ ^27^, ^32^, ^68^ ^]^

In addition, nanoparticular systems with significantly less than one plasma protein per nanoparticle, can also create the basis for new studies about the fate of nanoparticles, because modern analytical methods allow the determination of tiny changes happening on them. And thus, we may come close to the study of the live cycle of nanoparticles. In nature biological nanoobjects are constantly produced, but after some time also obsonicated and thereby designed for degradation. Such a situation was recently observed for a system of molecular brushes, which were functionalized with antibodies.^[^ ^101^ ^]^ The unmodified brushes had no sigh of protein corona formation. They circulated for a very long time in the body and were especially not taken up in the liver. But after conjugation of the antibodies (complete antibodies with F_c_‐unit) this changed and they were now taken up in the liver efficiently.^[^ ^101^ ^]^ In part, this may be a consequence of the F_C_‐units, but LC‐mass spectrometry showed that the brushes got also associated with some proteins of the complement system. Now this was still a very small amount of proteins (in fact 1 versus 2 antibodies per nanoparticle can already play a role in delivery),^[^ ^137^ ^]^ but such tiny changes can have an effect and assign the nanoparticle as an object, which should be degraded and removed from circulation. Thus, such particles with “nearly no” protein corona allow detailed studies on the “life‐circle” of nanoparticles.

### 4.2 Further Concepts to Prevent or “Steer” Protein Corona Formation

Generally, to protect the hard, inner part of a colloid against the access of proteins (entropical shielding), a permanently bound dense surface coating of hydrophilic polymers is sufficient (see Figure 1). And there are more general approaches to achieve this besides the concept to use block copolymers micelles as starting material or to work on grafting‐too or grafting from concepts (see end of session 3).

An alternative approach to coat colloids with a dense, uncharged, but hydrophilic corona to obtain “entropically shielded” structures, could be the use of amphiphilic structures with a very low CMC. Such amphiphiles can be made with amphiphilic block copolymers, e.g., with pHPMA and p‐laurylmethacrylate blocks.^[^ ^113^, ^128^ ^]^ The coating of the hydrophobic core can then be done by “miniemulsion technique in combination with solvent evaporation”.^[^ ^128^, ^138^ ^]^ In this case the hydrophobic part of the block copolymer orients toward the colloidal surface and the hydrophilic parts toward the outside (the plasma with its proteins). For this concept there are, however, no data concerning the achieved density of the hydrophilic chains on the colloid available yet. Shielding will only be effective, if the hydrophilic chains are packed dense enough and get stretched.^[^ ^96^, ^139^ ^]^ From the experiments performed in literature the concept seems to work, at least to some extent^[^ ^128^ ^]^ (no aggregation is observed for the coated polystyrene and polylactide colloids in contact with plasma and their size does not increase within accuracy), but the amount of proteins associated with the nanoparticles has not been quantified. Therefore, it cannot be differentiated, if only a very small amount of proteins is bound (tiny increase of the diameter) or really no proteins. This may depend on the coating process.

In addition, the kinetics during incubation with plasma proteins will play an important role in colloids covered with block copolymers, because amphiphilic block copolymers have a CMC (although it might be very low) and thus there are always some unimers in solution (see Figure 3). In this context it is interesting that recently it has been shown for non‐crosslinked block copolymer micelles with a low CMC (range from 10^−5^ to 10^−4 ^mg mL^−1^, that means small amount of unimer in equilibrium^[^ ^117^, ^140^, ^141^ ^]^) that they acquire more plasma proteins^[^ ^98^ ^]^ (although still not more than about 1 protein per micelle^[^ ^98^ ^]^) than the core crosslinked micelles, which cannot form an equilibrium with unimers. So any equilibrium, which modifies the surface with time, opens the possibility for adsorption of plasma proteins, as time goes on. Thus core crosslinked micelles are advantageous.^[^ ^71^, ^98^, ^99^ ^]^

It is, in this context, also interesting to consider the adsorption of amphiphilic plasma proteins as option to shield the particle surface form protein binding or recognition by the immune system.^[^ ^27^ ^]^ For some colloids,^[^ ^118^ ^]^ which are coated with a corona of hydrophilic polymers. The adsorption of certain plasma proteins leads to a “stealth like” nature afterwards.^[^ ^118^ ^]^ Such proteins are primarily apoproteins (ApoE), clusterine, and other amphiphilic proteins.^[^ ^80^ ^]^ They contain hydrophobic and hydrophilic parts and are used in the body, e.g., for the transport of lipid droplets. Similar results were reported after the adsorption of antibodies to hydrophobic colloids.^[^ ^65^ ^]^ So the situation might be ‐from a physico‐chemical standpoint‐ similar to the adsorption of artificial amphiphilic block copolymers.^[^ ^128^ ^]^ The hydrophobic parts of these proteins can interact and adsorb to hydrophobic patches on the colloids. But thereby they will shield these structures via the hydrophilic parts of the protein. Thus, certain proteins may ‐after adsorption‐ offer a physico‐chemical possibility for a shielding of colloids against further adsorption of more plasma proteins. Such effects are also known from planar hydrophobic surfaces, where it is, e.g., possible to reduce the formation of a protein layer by a pre‐incubation with serum albumin.^[^ ^12^, ^142^ ^]^ However, there are no data available with respect to long term or in vivo stability.

This concept has been widely expanded into a “steering” of the protein‐corona^[^ ^27^ ^]^ by a pre‐adsorption of special proteins by Mailänder and Landfester.^[^ ^65^, ^102^, ^108^ ^]^ It has the potential to become important for useful pharma transport systems, if the long term stability problem can be solved. Work on model systems (no potential as transporters) may provide lots of data, but it is questionable if the obtained knowledge is transferable to other API transport systems with potential for clinical translation. This applies especially to poorly shielded nanoparticles (e.g., polystyrene colloids), which acquire a massive protein corona.

### 4.3 Further Materials for the Formation of a Hydrophilic Protecting Polymer Shell

As there are different ways to coat the nanoparticle with a dense hydrophilic shell of polymers (graft‐to, grafting from, or simple adsorption of amphiphilic structures) it seems promising to study different polymers for this purpose. Given the tendency to form highly hydrated systems,^[^ ^82^, ^83^, ^91^ ^]^ zwitterionic structures look especially promising in this context. Zwitterionic polymers show no net charge (important for lang range ionic interactions) but are able to coordinate water molecules in the vicinity of the charged groups very effectively. But as recently pointed our,^[^ ^91^ ^]^ it is essential to control their internal structure, because otherwise their positive and negative charges may compensate each other too perfectly, which can reduce their solubility and the permeation of water.

## 5 Conclusion

The question about the significance (and role) of a protein corona on different classes of nanoparticles cannot be answered in a “general sense”. This is not due to an inherent issue with the nanodimensions of the particles. Instead, it arises from the nature of the nanosystem (nanoparticles and plasma proteins, forming a mixed colloidal system) having an especially large surface area. Thus the system is ‐to some extend‐ instable^[^ ^7^, ^9^, ^74^ ^]^ and the deposition of some plasma proteins ‐with time‐ on the nanoparticles is “likely”. As a result, the popular assertion that any nanoparticle forms a protein corona upon contact with blood plasma is generally true for nearly all nanoparticles. However, some nanoparticles designed for nanomedicine applications do not absorb any proteins and, therefore, do not form a protein corona. This occurs for well‐understood reasons: the cores of these nanoparticles are coated with a dense layer of strongly hydrated, hydrophilic polymers, which effectively keep plasma proteins away. This is achieved through a combination of a bound water layer and entropic shielding.

The next question is: How important is the formation of a protein corona. Now, corona formation can be studied, and the results provides certain insights into highly complex systems. Work on this topic accounts for about 1.500 publications up to the middle of 2022.^[^ ^7^ ^]^ It excludes, however, mostly the question, if corona formation is unavoidable. In our opinion, the real significance of corona formation has to come, however, from the interest to use nanoparticles in the broad field of nanomedicine. And here, the formation of a protein corona ‐at least in cases, when corona formation is strong‐ is disadvantageous. That is because nanomedicine wants to use nanoparticles in patients differing ‐to a certain extend‐ in serum protein composition. A central idea is thereby to decouple the pharmacological action of an active compound from its body distribution (e.g., to bring drugs into a tumor, or to bring an antigen and adjuvant to immune cells in the skin, spleen or lymph nodes. The formation of a thick protein corona will, however, strongly modify the body distribution, because it causes PK variation in patients. Under this aspect nanoparticles with little or no protein corona formation are advantageous. Nanoparticles, without protein corona are also ideal for biomedical approaches to activate the immune system since they avoid coprocessing of adsorbed proteins.^[^ ^25^ ^]^ The procession of endogeneous proteins (as part of the protein corona) may even be the starting point of auto‐immune response. It is interesting ‐in this aspect‐ that such immune responses have been observed.

In addition, nanoparticles with no protein corona formation can be made systematically. They can be synthesized from core‐crosslinked block copolymer micelles, but also by grafting‐from or grafting‐to methods. It may also be that a direct deposition of amphiphilic blockcopolymers gets possible. In this case, however, nothing is known yet on the long‐term stability of the system, which may just rearrange in an unfavorable way with time. In addition, more hydrophilic, strongly hydrated polymers are ‐generally‐ access able to prepare the hydrophilic shells, which prevent the deposition of the plasma proteins. On the other hand, designing nanoparticles that attract specific, ideally disease related proteins holds the enormous potential to boost early detection of diseases.

## Conflict of Interest

The authors declare no conflict of interest.
